# Expanding the Toolbox of Biomedical HIV Prevention Options Through Innovative Design

**DOI:** 10.3390/pharmaceutics17060742

**Published:** 2025-06-05

**Authors:** J. Gerardo García-Lerma

**Affiliations:** Laboratory Branch, Division of HIV Prevention, National Center for HIV, Viral Hepatitis, STD, and TB Prevention, Centers for Disease Control and Prevention, Atlanta, GA 30329, USA; jng5@cdc.gov

Research on biomedical HIV prevention has shown that no single product can meet the needs and preferences of all individuals [1]. Despite the increasing availability of options for HIV pre-exposure prophylaxis (PrEP), overall coverage remains suboptimal [2]. Daily oral pre-exposure prophylaxis (PrEP) with emtricitabine (FTC) and tenofovir disoproxil fumarate (TDF) or tenofovir alafenamide (TAF) is highly effective, yet adherence to a daily pill poses challenges for some individuals [3]. Long-acting injectable drug formulations such as cabotegravir (CAB) and lenacapavir can help address adherence challenges and offer a discreet option with extended dosing intervals. However, the frequency of injections and required clinical visits may deter some users [4]. For women, a 1-month intravaginal ring delivering dapivirine provides an alternative PrEP method. Nevertheless, preferences for rings vary across age groups and settings [5,6]. An on-demand PrEP option with FTC/TDF is available for men who have sex with men, offering a suitable option for individuals with infrequent, unplanned sexual activity or those who prefer not to use daily or injectable PrEP [7,8]. However, this approach follows a complex 2-1-1 schedule, requiring two FTC/TDF pills before sex, followed by one pill at 24 h and another at 48 h post-sex, which can be challenging to maintain.

This Special Issue of *Pharmaceutics*, “HIV/AIDS Prevention Formulation Design and Optimization and Its Pharmacokinetic-Pharmacodynamic Evaluation” (https://www.mdpi.com/journal/pharmaceutics/special_issues/1STR546UJF, accessed on 18 April 2025), highlights several innovative strategies for delivering PrEP and expanding the toolbox of available options. Several studies focus on long-acting or ultra-long-acting PrEP, including monoclonal antibodies and new drug formulations and delivery methods. As highlighted in a review article by Nayan et al., (contribution 1) ideal candidates for long-acting PrEP should consider key attributes including long dosing intervals, a short pharmacokinetic (PK) tail after treatment discontinuation, and factors such as cost, storage, and transportation requirements. Curley et al. (contribution 2) describe a semi-solid prodrug nanoparticle formulation of FTC, highlighting how this strategy has the potential to extend the half-life of water-soluble drugs and increase the availability of long-acting products. In two related studies, Kinsale et al. (contribution 3) and Daly et al. (contribution 4) explore reservoir-style poly (ɛ-caprolactone) biodegradable implants for the delivery of islatravir either alone or in combination with the hormonal contraceptive etonogestrel. This multipurpose prevention technology could offer women dual protection in a single product, addressing reproductive health concerns while promoting PrEP uptake. Young et al. (contribution 5) characterize the pharmacokinetics, biodegradation, and removability of poly(lactic-co-glycolic acid)-based in situ forming implants (ISFIs) containing CAB. In early preclinical studies, ISFIs were well tolerated, provided ultra-long-acting protection against repeated simian HIV (SHIV) exposure in macaques, and could be co-formulated with hormonal contraceptives for broader prevention benefits [9,10]. Mayer et al. (contribution 6) report on the PK of broadly neutralizing monoclonal antibodies (mAb) that incorporate LS mutations (M428L/N434S) in the Fc region to extend the durability for PrEP or treatment. Using population PK models and data from 16 clinical trials with 5 different LS and parental versions of mAbs, they show how the LS mutations improve PK by increasing elimination half-life, area under the curve value, and serum concentrations. These characteristics may offer extended durability for PrEP or treatment, potentially reducing dosing frequency.

Approaches that rely on more frequent dosing or on-demand use are also discussed in this Special Issue. Kinvig et al. (contribution 7) and Vora et al. (contribution 8) highlight the promise of microarray patches (MAPs) to deliver HIV PrEP or treatment. MAPs present a promising drug delivery platform that is user-friendly and suitable for self-administration. Kinvig et al. (contribution 7) use physiologically based pharmacokinetic modeling to investigate the PK of MAPs delivering CAB, defining optimal dosing strategies to maintain plasma CAB concentrations above protective thresholds for a week. Vora et al. (contribution 8) address the complexity of drug delivery via MAPs, emphasizing the importance of considering both microneedle designs and drug formulations to achieve effective delivery of CAB or other antiretroviral drugs. While questions remain regarding the required patch size to deliver appropriate CAB concentrations, these studies set the stage for new patch designs that may leverage more potent drugs requiring lower loading doses. An article by Peet et al. (contribution 9) highlights the potential of topical inserts—small, tablet-like products that can be discreetly packaged and used on demand for rectal and vaginal protection against HIV. Discreet design and portability may help increase accessibility and user preference. In preclinical studies, inserts containing TAF and elvitegravir were safe, effective, and versatile, offering potential for both pre- and post-exposure application.

Given the dynamic nature of individual preferences and circumstances, continued development of new PrEP options is essential to achieve global HIV prevention targets. Studies such as the ones described in this Special Issue of *Pharmaceutics* may help prioritize products that have the highest potential for clinical advancement, expand the range of available methods, and ultimately enhance overall PrEP uptake.
